# Supplementary material for: Crosstalk between the chloroplast protein import and SUMO systems revealed through genetic and molecular investigation in Arabidopsis
Source: eLife. 2021 Sep 2;10:e60960. doi: 10.7554/eLife.60960 (PMC8497055; doi:10.7554/eLife.60960)
Supplement: Transparent reporting form [file elife-60960-transrepform1.docx]

***eLife’s* transparent reporting form**

We encourage authors to provide detailed information *within their submission* to facilitate the interpretation and replication of experiments. Authors can upload supporting documentation to indicate the use of appropriate reporting guidelines for health-related research (see [EQUATOR Network](http://www.equator-network.org/%20)), life science research (see the [BioSharing Information Resource](https://biosharing.org/)), or the [ARRIVE guidelines](http://www.plosbiology.org/article/info:doi/10.1371/journal.pbio.1000412) for reporting work involving animal research. Where applicable, authors should refer to any relevant reporting standards documents in this form.

If you have any questions, please consult our Journal Policies and/or contact us: [editorial@elifesciences.org](mailto:editorial@elifesciences.org).

**Sample-size estimation**

- You should state whether an appropriate sample size was computed when the study was being designed
- You should state the statistical method of sample size computation and any required assumptions
- If no explicit power analysis was used, you should describe how you decided what sample (replicate) size (number) to use

Please outline where this information can be found within the submission (e.g., sections or figure legends), or explain why this information doesn’t apply to your submission:

No explicit power analysis was used. We typically performed at least three biological replicates for each experiment, unless otherwise noted in the figure legends or the Materials and methods section. The phenotypes of the plants were highly consistent, with minimal variation.

**Replicates**

- You should report how often each experiment was performed
- You should include a definition of biological versus technical replication
- The data obtained should be provided and sufficient information should be provided to indicate the number of independent biological and/or technical replicates
- If you encountered any outliers, you should describe how these were handled
- Criteria for exclusion/inclusion of data should be clearly stated
- High-throughput sequence data should be uploaded before submission, with a private link for reviewers provided (these are available from both GEO and ArrayExpress)

Please outline where this information can be found within the submission (e.g., sections or figure legends), or explain why this information doesn’t apply to your submission:

This information can be found within the relevant figure legends and in the Materials and methods section.

Biological replicates involved measurements or samples taken from distinct plants or samples, whereas technical replicates involved measurements or procedures taken or repeated multiple times using the same plant or sample.

The number of biological replicates per sample is displayed above each bar of each graph.

We removed one outlier when processing the data. This was encountered during the quantification of the immunoblots that appear in Figure 1I and Source data 1. In this case, the Toc75 band was unusually weak in the wild-type sample. There was an obvious qualitative difference between this band and the other Toc75 wild-type bands in the companion experiments. We marked this panel with asterisks to indicate that it was not used in the quantification.

No high-throughput sequence data were generated in this work.

**Statistical reporting**

- Statistical analysis methods should be described and justified
- Raw data should be presented in figures whenever informative to do so (typically when N per group is less than 10)
- For each experiment, you should identify the statistical tests used, exact values of N, definitions of center, methods of multiple test correction, and dispersion and precision measures (e.g., mean, median, SD, SEM, confidence intervals; and, for the major substantive results, a measure of effect size (e.g., Pearson's r, Cohen's d)
- Report exact p-values wherever possible alongside the summary statistics and 95% confidence intervals. These should be reported for all key questions and not only when the p-value is less than 0.05.

Please outline where this information can be found within the submission (e.g., sections or figure legends), or explain why this information doesn’t apply to your submission:

The statistical analysis information can be found in the relevant figures and legends, and in the Materials and method section. The information includes test type, the exact number of biological replicates, and the *p* values generated by each comparison. In addition, all individual datapoints are displayed in each graph.

(For large datasets, or papers with a very large number of statistical tests, you may upload a single table file with tests, Ns, etc., with reference to sections in the manuscript.)

**Group allocation**

- Indicate how samples were allocated into experimental groups (in the case of clinical studies, please specify allocation to treatment method); if randomization was used, please also state if restricted randomization was applied
- Indicate if masking was used during group allocation, data collection and/or data analysis

Please outline where this information can be found within the submission (e.g., sections or figure legends), or explain why this information doesn’t apply to your submission:

For physiological experiments, plants of the different genotypes were randomly selected to mitigate potential variation due to environmental factors. Restricted randomization was not applied. For molecular and cell biological experiments, blinding was not required because the results of such measurements are not affected by knowledge of sample identities. Masking was not used during data collection.

**Additional data files (“source data”)**

- We encourage you to upload relevant additional data files, such as numerical data that are represented as a graph in a figure, or as a summary table
- Where provided, these should be in the most useful format, and they can be uploaded as “Source data” files linked to a main figure or table
- Include model definition files including the full list of parameters used
- Include code used for data analysis (e.g., R, MatLab)
- Avoid stating that data files are “available upon request”

Please indicate the figures or tables for which source data files have been provided:

The unprocessed membrane images supporting each immunoblot have been provided in Source data 1.
